# Novel B cell-dependent multiple sclerosis model using extracellular domains of myelin proteolipid protein

**DOI:** 10.1038/s41598-020-61928-w

**Published:** 2020-03-19

**Authors:** Alexander W. Boyden, Ashley A. Brate, Nitin J. Karandikar

**Affiliations:** 1 Department of Pathology, University of Iowa Health Care, Iowa City, IA USA; 2 Iowa City Veterans Affairs Medical Center, Iowa City, IA USA

**Keywords:** Autoimmunity, Immunology, B cells

## Abstract

Therapeutic success of B cell-targeting approaches in multiple sclerosis (MS) has intensified research into the pathogenic and regulatory roles these cells play in demyelinating disease. Dissecting the function of B cells in the MS mouse model experimental autoimmune encephalomyelitis (EAE) is largely confined to induction with either the myelin oligodendrocyte glycoprotein epitope MOG_35–55_ or the full-length recombinant human MOG protein, the latter representing the most-used B cell-dependent EAE model. There is a clear need to investigate B cell function in additional myelin antigen contexts. Unlike MOG_35–55_, where lack of B cells yields more severe disease, we show here that the immunodominant myelin proteolipid protein epitope (PLP_178–191_) elicited identical EAE in WT and μMT mice, suggesting an absence of B cell engagement by this peptide. We hypothesized that a longer PLP antigen may better engage B cells and designed a peptide encompassing the extracellular domains (ECD) of PLP. We demonstrate here that PLP_ECD_-immunized B cell-deficient mice failed to exhibit EAE. In contrast, PLP_ECD_ induced EAE not only in WT mice, but in B cell-sufficient mice incapable of secreting antibodies, suggesting a predominant antigen presentation role. These results establish a novel, efficient B cell-dependent EAE model.

## Introduction

Recent clinical trials involving the targeted deletion of B cells^1–3^ have reinvigorated intense interest in the role of this lymphocyte in immune-mediated demyelinating diseases such as multiple sclerosis (MS). It is essential to have multiple models, whereby various angles of B cell biology (such as their potential pathogenic or regulatory roles) can be deciphered in the context of disease. Historically, myelin oligodendrocyte glycoprotein (MOG) sequence 35–55 has been used to great success in inducing demyelinating disease in WT B6 mice^4,5^, where knockout capabilities and genetic tools like the 2D2 system (mice harboring a TCR transgene specific for the MOG_35–55_ epitope) are available^6^.

B cell studies in MOG-induced demyelinating disease have led to a contradictory literature, in that there is evidence for both pathogenic and regulatory roles, as well as models that require and yet do not require the presence of B cells for EAE. These discrepancies can largely be explained by differences in model type, animal strain, and inciting antigen used. For instance, antibody titers and B cell numbers in the cerebrospinal fluid (CSF) have been shown to correlate with disease severity in mice and humans^7–10^. Likewise, full length recombinant human MOG (hMOG)-reactive serum transfers from B6 mice have been shown to cause disease in healthy animals^11^. Decreased demyelination in B cell-deficient versus WT B6 mice has been observed (yet both exhibited disease)^12^ while other data demonstrate that induction of EAE failed altogether in B cell-deficient mice^13^. Interestingly, these studies utilized rodent MOG versus human MOG, respectively. It has further been demonstrated that deleting B cells from MOG_35–55_ EAE mice during peak disease ameliorates clinical symptoms^14^. In addition to pathogenic antibody studies, B cells have been shown to contribute to rodent MOG-induced EAE in C3H mice as well as hMOG-induced EAE in B6 mice by reactivating CD4 T cells in the CNS through a likely antigen presentation function^15–18^. These reports of B cell pathogenicity notwithstanding, it has also been demonstrated that B cell-deficient mice cannot recover from myelin basic protein-induced EAE disease in B10.PL mice^19^ and have a quicker and more robust MOG_35–55_ and rodent MOG disease onset compared to their WT B6 counterparts^20,21^. Further, deletion of B cells prior to MOG_35–55_ disease onset led to exacerbated disease^14^, adding to studies attributing regulatory function to B cells during EAE.

These data clearly suggest that B cell subsets have varying functional capacities (IL-10-producing Bregs^20^ versus CD4 T cell-reactivating CNS B cells^15^, for example). However, the general discrepancies seen in these reports, particularly regarding experiments involving B cell-deficient mice, can be explained by differences in the length of MOG antigen used for immunization (short peptide versus full-length recombinant protein), and the origin of MOG antigen (rodent versus human). It turns out that a single amino acid at position 42 (serine in the rodent, proline in the human) in the MOG sequence^22,23^ can have striking consequences for the role of B cells in MOG-induced EAE models. Ultimately, it is understood that recombinant hMOG is necessary to induce a robust B cell-dependent mouse model of MS^13,22,23^. Yet it is still unclear whether this is due to pathogenic antibody production or requirement for antigen processing and presentation. In addition, production of the hMOG protein is cumbersome and represents a significant rate-limiting step in the study of B cell functionality *in vivo* during demyelinating disease. Finally, heavy reliance on a single model may bias our understanding of the role of these cells in complex human disease.

Investigating the role of B cells in myelin proteolipid protein (PLP)-induced EAE models is needed. PLP is highly conserved, where complete amino acid sequence homology is shared between mice and humans, and is very abundant in the CNS, comprising 50% of total myelin protein^24^. Indeed, understanding immune responses in this context is extremely relevant and may provide insights into the pathogenicity and regulation of MS. Whereas PLP-targeted responses are a focus in the relapsing-remitting SJL mouse model of EAE^25,26^, this protein or its peptides are underutilized in the context of B6 models, where there is a greater availability of genetic tools to dissect the function of various cells and molecules. We have recently utilized PLP_178–191_-induced EAE in B6 mice to demonstrate the robust disease regulatory role of PLP_178–191_-induced CD8 T cell responses^27–30^. We therefore decided to focus on PLP-induced EAE to develop a model wherein B cell function could also be delineated. Interestingly, our findings here suggest that B cells are ancillary during the immunodominant PLP_178–191_-incduced form of EAE in B6 mice. We hypothesized that designing a longer peptide may provide a processable antigen for B cells to engage and present to T cells, thus alleviating the need for intricate whole protein production methodology. Here, we utilized a novel designed peptide (PLP_ECD_, an 83-mer that incorporates the extracellular sequences of PLP while excluding positions buried within or just proximal to transmembrane regions) to test B cell-dependency in the context of PLP-induced demyelinating disease in B6 mice. We offer an efficient new mouse model suitable to investigating potential roles for B cells in EAE.

## Materials and methods

### Mice

C57BL/6 J and μMT mice were purchased from Jackson Laboratories (Bar Harbor, ME). AID−/−μS−/− mice were kindly provided by Drs. Frances Lund and Troy Randall (University of Alabama, Birmingham) and Dr. Tasuku Honjo (Kyoto University, Japan). All mice were kept in barrier rooms at the University of Iowa Animal Care Facility under 12-hour light/dark cycle, fed ad libitum, and humanely cared for and studied as approved by the University of Iowa’s Institutional Animal Care and Use Committee and in accordance with the National Institutes of Health guide for the care and use of Laboratory animals (NIH Publications No. 8023, revised 1978).

### Peptides

Myelin peptides PLP_178–191_ (NTWTTCQSIAFPSK), MOG_35–55_ (MEVGWYRSPFSRVVHLYRNGK), and control peptide OVA_323–339_ (ISQAVHAAHAEINEAGR) were purchased from GenScript (Piscataway, NJ). Myelin peptide PLP_36–58;179–238_ or “PLP_ECD_” (HEALTGTEKLIETYFSKNYQDYETWTTCQSI AFPSKTSASIGSLCADARMYGVLPWNAFPGKVCGSNLLSICKTAEFQMTFHL) was purchased from Thermo Pierce Custom Peptides (Waltham, MA).

### EAE immunization and scoring

As described previously^27–30^, mice were immunized s.c. on d0 in the right and left flank with 100ug of PLP antigen emulsified 1:1 volume in complete Freund’s adjuvant supplemented with 4 mg/ml *Mycobacterium tuberculosis* (CFA; Becton Dickinson, Franklin Lakes, NJ), followed by 250 ng pertussis toxin (PTx; List Biologicals, Campbell, CA) i.p. on days 0 and 2. Clinical scores were recorded in a blinded manner by ascending hind limb paralysis: 0, no symptoms; 1, loss of tail tonicity; 2, partial hind limb weakness; 3, partial hind limb paralysis; 4, complete hind limb paralysis; 5, moribund or death.

### Delayed-type hypersensitivity (DTH)/ear swelling assays

As described previously^30^, ear pinnae of briefly anesthetized (isoflurane USP, Clipper Distributing, St. Joseph, MO) ear pinnae of mice were injected with 150ug PLP_178–191_, PLP_ECD_, or OVA_323–339_ in 15 µl volume PBS at the indicated timepoints post-immunization with a 30 G needle from a 1cc syringe. Additional control ears received 15 µl PBS alone. Delta ear swelling was measured in a blinded manner with an engineer’s micrometer (Mitutoyo USA, Aurora, IL) and calculated by ear thickness (mm) at 48 h minus thickness at 0 h.

### Statistical analysis

When comparing two groups, data were analyzed using the Welch’s *t*-test. For multiparametric data, the two-way ANOVA test with Tukey post-test was performed. All graph production and statistical analyses were done using GraphPad Prism software (La Jolla, CA).

## Results and Discussion

### B cells are not required for EAE induction or progression in PLP_178–191_-immunized B6 mice

**Figure 1:**
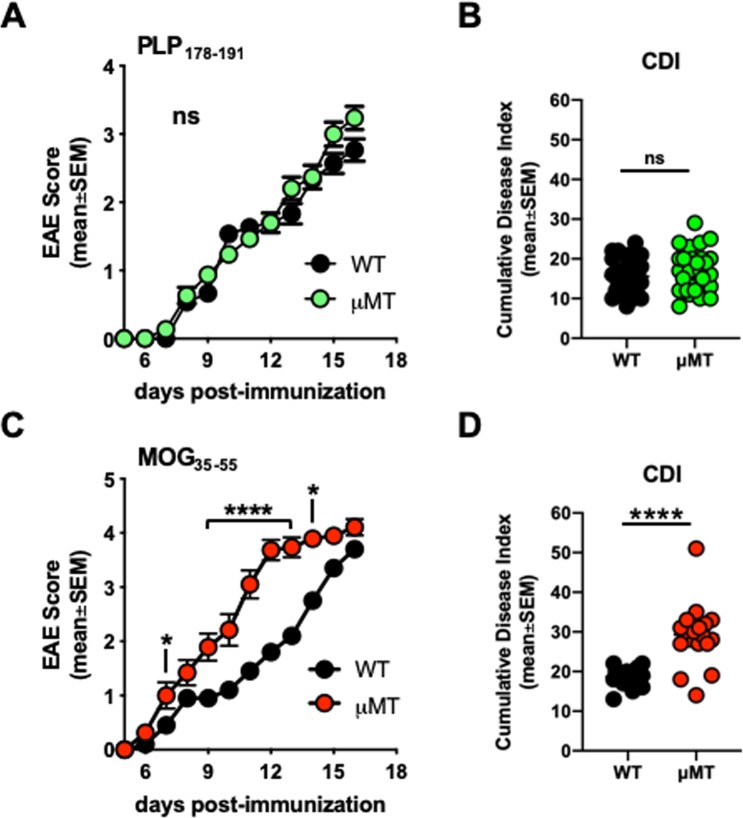
B cells are not required for EAE induction or progression in PLP_178–191_-immunized B6 mice. WT or μMT mice were immunized s.c. with 100ug PLP_178–191_/CFA or MOG_35–55_/CFA on day 0 and given 250 ng pertussis toxin i.p. on days 0 and 2. (**A,C**) Clinical disease scores. (**B,D**) Cumulative disease index (CDI; measure of disease severity) depicts the summation of paralysis scores over time for each individual mouse in all groups. PLP_178–191_ groups: n = 30 WT; n = 30 μMT. MOG_35–55_ groups: n = 20 WT; n = 19 μMT. Data is representative of three independent experiments. ns = not significant; *p < 0.05; ****p < 0.0001.

Prior studies have shown that B cells may play a protective role in MOG_35–55_-induced EAE^14,20,21,31,32^. However, it is not clear whether B cells play any role in other peptide-induced EAE B6 models and whether this phenomenon is generalizable. We therefore immunized WT B6 and B cell-deficient (μMT) mice with PLP_178–191_/CFA and monitored paralytic disease. We observed that B cell-deficient mice immunized with PLP_178–191_/CFA exhibited unaltered EAE progression, similar to that observed in WT mice (Fig. 1A). Cumulative disease index (CDI, or the summation of disease scores over time per mouse) was also not different (Fig. 1B). In contrast, MOG_35–55_/CFA-immunized B-cell deficient mice exhibited faster EAE kinetics and showed more severe disease (Fig. 1C,D), and is in accordance with previous literature^20^. This is also in contrast to observations in a susceptible BALB/c model, where B cells may play a regulatory role in the context of PLP_180–199_ or PLP_185–206_ peptide-induced EAE^33^. These data indicate that B cell engagement is suboptimal in PLP_178–191_-immunized B6 mice, and suggests that a short peptide-length PLP antigen is insufficient to drive either protective or pathogenic B cell responses *in vivo* in mice of this genetic background. These results also illustrate that it is not yet clear which PLP domains drive which B cell characteristics during disease.

### Designed 83-mer peptide encompassing the extracellular domains of myelin proteolipid protein (PLP_ECD_) elicits immune responses *in vivo*

**Figure 2:**
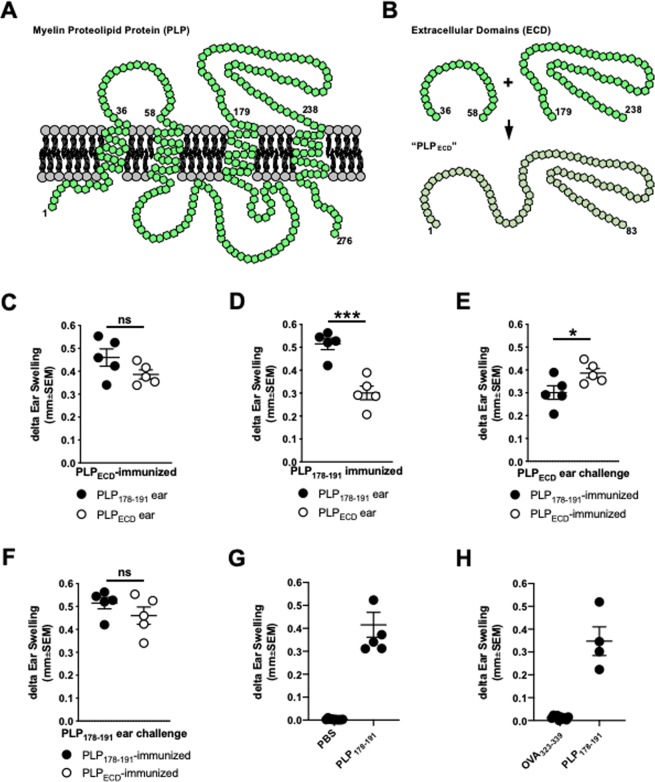
Designed 83-mer peptide encompassing the extracellular domains of myelin proteolipid protein (PLP_ECD_) elicits immune responses *in vivo*. (**A**) Multi-pass transmembrane orientation of PLP. Diagram is inspired by Hudson, 2004 (ref. ^24^) and Appikatla *et al*., 2014 (ref. ^34^). (**B**) Sequences corresponding to the extracellular domains of PLP were utilized to design a novel 83-mer polypeptide. WT B6 mice were immunized s.c. with either 100ug PLP_ECD_/CFA or PLP_178–191_/CFA on day 0. (**C,D**) On day14, groups of PLP_ECD_- and PLP_178–191_-immunized mice were challenged with PLP_178–191_ or PLP_ECD_ in the right and left ear pinnae of each mouse, respectively. Ears were measured at 48 h. (**E,F**) Reanalysis of data in (**C,D**) comparing similar ear challenges between two immunization groups (ie. white symbols in (**C,D**) are compared in (**E**) and black symbols in (**C,D**) are compared in (**F**). (**G,H**) Control experiments depicting 48 h ear swelling elicited by the indicated antigen challenges in mice immunized with PLP_178–191_ 14 days prior. ns = not significant; *p < 0.05; ***p < 0.001.

It has been demonstrated that changes in MOG_35–55_/CFA-induced disease kinetics within μMT mice are due to the absence of naturally occurring IL-10-producing B1 Bregs^14^. This contrasts with switched high affinity follicular B cells, which may be pathogenic later in MOG_35–55_-induced disease^14^. Interestingly, in order to induce a B cell-dependent EAE model, one must immunize mice with full length recombinant hMOG protein rather than a smaller – albeit immunodominant – peptide (MOG_35–55_)^13^. Given the seemingly ancillary nature of B cells in PLP_178–191_-immunized mice (Fig. 1A,B), we hypothesized that a longer peptide (perhaps mimicking full length protein in a limited way) may provide more epitopes and structure with which B cells could potentially engage and process for presentation. We therefore designed a novel peptide encompassing both of two extracellular PLP domains, termed “PLP_ECD_”. This is depicted in Fig. 2 in a diagram inspired by^34^ and^24^. PLP is an extremely hydrophobic multi-pass transmembrane protein (Fig. 2A). We therefore chose to incorporate extracellular sequences that excluded positions buried within, or just proximal to, the transmembrane region of the polypeptide structure while still containing as much of the immunodominant PLP_178–191_ epitope as possible (Fig. 2B). The final designed sequence was therefore an 83-mer spanning most of the two extracellular domains, including positions 179–191 from the immunodominant epitope (Fig. 2B).

Given that EAE is an immune-mediated model of demyelination^35,36^, we next determined the immunogenicity of PLP_ECD_ peptide by testing whether it could induce cellular immune responses *in vivo*. Type IV delayed-type hypersensitivity (DTH) assays have been utilized for decades as a measure of non-humoral, T cell-driven inflammation *in vivo*, and furthermore been used to read out immune responses to CNS myelin antigens^30,37–41^. Here, we performed DTH experiments as described previously^30^. Briefly, this involved making mice immune through s.c. flank injection with myelin peptide/CFA and subsequently challenging ear pinnae of immune mice with the same or similar myelin peptide (without CFA) or PBS alone (control) injection two weeks later. Using an engineer’s micrometer, measuring the ear thickness of injected ears at 48 h compared to ear thickness at a background 0 h measurement, one can identify “delta ear swelling” as a readout of the DTH reactions occurring in the challenged, immune animals. Specifically, individual mice were challenged with PLP_178–191_ in the right ear pinna and PLP_ECD_ in the left ear pinna 14 days following either PLP_178–191_- or PLP_ECD_-immunization. Swelling was measured at 48 h post-ear challenge. PLP_ECD_-immunized mice exhibited robust ear swelling when challenged with either PLP_ECD_ or PLP_178–191_ (Fig. 2C), indicating that PLP_ECD_ is sufficiently engaged, processed, and presented in these mice. As expected, PLP_178–191_-immunized mice exhibited robust swelling in PLP_178–191_-challenged ears (Fig. 2D, black symbols). PLP_ECD_ also induced DTH responses in PLP_178–191_-immunized mice (Fig. 2D, white symbols), albeit at lower levels than in PLP_ECD_-immunized mice (Fig. 2E). This may be due to availability of epitopes on a per cell basis *in vivo*. PLP_178–191_-driven DTH was similarly elicited in both PLP_178–191_- and PLP_ECD_-immune mice (Fig. 2F), indicating a robust induction of responses to this pathogenic epitope. Importantly, DTH does not develop when challenging ear pinnae of PLP_178–191_-immune mice with a control PBS alone injection (Fig. 2G) or with a non-cognate ovalbumin peptide (Fig. 2H). Collectively, these results demonstrate the immunogenicity of PLP_ECD_.

### PLP_ECD_ induces B cell-dependent EAE in B6 mice

**Figure 3:**
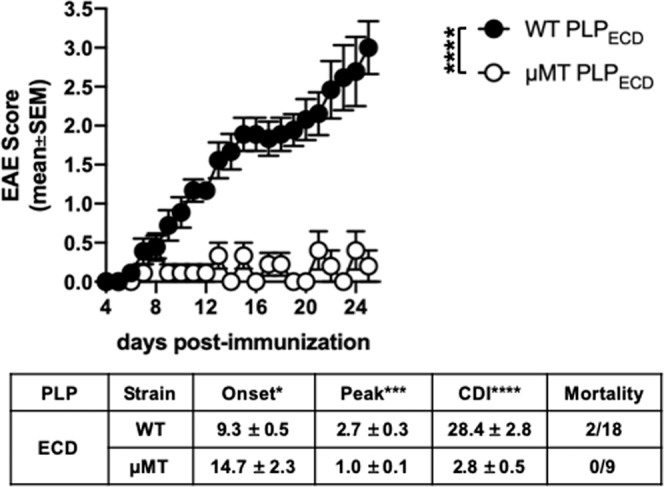
PLP_ECD_ induces B cell-dependent EAE in B6 mice. WT or μMT mice were immunized s.c. with 100ug PLP_ECD_/CFA on day 0 and given 250 ng pertussis toxin i.p. on days 0 and 2. Clinical disease scores were monitored and EAE parameters were analyzed (depicted in table as mean values ± standard error of the mean). Data presented includes scores from two to three independent experiments. ns = not significant; *p < 0.05; ***p < 0.001; ****p < 0.0001.

To test whether PLP_ECD_ could successfully induce EAE, and to determine whether PLP_ECD_ could successfully engage B cells (either in a pathogenic or regulatory role), WT and B cell-deficient (µMT) B6 mice were actively immunized with PLP_ECD_/CFA s.c. on day 0 along with i.p. injections of pertussis toxin on days 0 and 2. Clinical disease scores were monitored over time. Repeated experimentation composited in Fig. 3 demonstrates that EAE was indeed robustly induced in WT B6 mice following immunization with PLP_ECD_ (Fig. 3, black symbols). Intriguingly, µMT mice exhibited minimal disease induction (Fig. 3, white symbols). The two groups of mice were statistically different not only when comparing raw paralysis scores, but also with respect to day of onset, peak score of disease, and CDI (Fig. 3, EAE parameter table). These results ultimately suggest that PLP_ECD_ is a potent inducer of encephalitogenic responses in B6 mice, and that PLP_ECD_-driven EAE disease is B cell-dependent.

It is important to acknowledge that μMT mice could have compromised priming dynamics due to altered lymphoid and splenic architecture, resulting in priming loss brought about in a somewhat lymphocyte-extrinsic manner. However, this is not the case in our models, where despite the complications μMT mice present in these respects, Fig. 1 clearly shows equivalent EAE between μMT and WT mice when immunized with PLP_178–191_. Likewise, even in the face of B cell deficiency, PLP_178–191_-immune μMT and WT mice show similar PLP_178–191_-driven DTH reactions over background (Supplementary Fig. 1). This suggests that T cell priming in the context of PLP_178–191_ is not only largely unaffected, but is dominated by antigen presenting cell populations other than B cells (eg. dendritic cells). Priming is likely much different in the context of the larger antigen PLP_ECD_. As demonstrated in Fig. 3, lack of B cells results in loss of robust EAE induction. Further, in the context of PLP_ECD_ immunization, lack of B cells results in a diminished PLP_178–191_-driven DTH reaction, similar to background levels (Supplementary Fig. 1). This result indicates that unlike PLP_178–191_ immunization, which is largely a B cell-independent priming, PLP_ECD_ requires B cells as a critical antigen-presenting cell for optimal T cell priming, and that other antigen presenting cells like dendritic cells are unable to fully liberate and present the bulk of the immunodominant epitope from the novel peptide. Future studies of PLP_ECD_-induced germinal centers to establish how B cells interact with T cells in this model will be illuminating.

### B cell-dependency in PLP_ECD_-driven EAE disease in B6 mice occurs irrespective of antibody production

**Figure 4:**
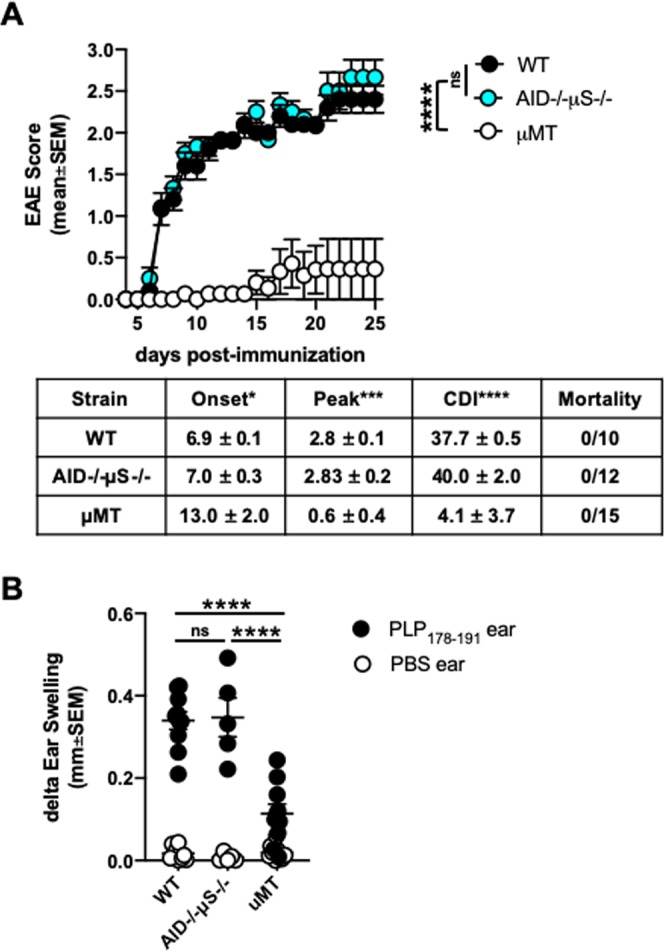
B cell-dependency in PLP_ECD_-driven EAE disease in B6 mice occurs irrespective of antibody production. (**A**) WT, μMT, and AID−/−μS−/− mice were immunized s.c. with 100ug PLP_ECD_/CFA on day 0 and given 250 ng pertussis toxin i.p. on days 0 and 2. Clinical disease scores were monitored and EAE parameters were analyzed (depicted in table as mean values ± standard error of the mean). (**B**) On day 23 post-immunization, groups of mice in **A** were challenged with PLP_178–191_ or PBS alone in right and left ear pinnae, respectively. Ears were measured at 48 h. ns = not significant; *p < 0.05; ***p < 0.001; ****p < 0.0001.

Functionally, the dependency of B cells in this model likely rests on antigen-presentation and/or pathogenic antibody production. It is known that oligoclonal switched IgG antibody bands within the CSF, along with B cells, are an indicator of MS disease^9^. Antibodies have also been shown to be pathogenic in some models of EAE. Antigen presentation function may also be important for B cell pathogenicity, as B cells have been shown to reactivate encephalitogenic CD4 T cells in the CNS of EAE mice^15–18^. Clearly, B cells play a role in MS as recent clinical trials have shown success with depleting B cells^1–3^. However, these depletion strategies target CD20+ B cells and leave behind antibody-producing plasma cells and plasma blasts. Given these data, in addition to those seen here, we wondered whether antigen presentation, rather than antibody production, explains the B cell-dependency of EAE induction in this model, perhaps reflecting their role in treated MS patients. To formally test this, we utilized AID−/−μS−/− mice^42^, which have a full complement of B cells, but lack the ability to secrete antibodies. We hypothesized that unlike μMT mice, which lack mature B cells and do not exhibit EAE disease, AID−/−μS−/− mice would be susceptible to EAE induction with PLP_ECD_. Indeed, upon immunization, AID−/−μS−/− exhibited a similar disease course compared to WT mice (Fig. 4A). And as expected given the data in Fig. 3, μMT mice showed little EAE disease in comparison to their B cell-sufficient counterparts (Fig. 4A). In analyzing EAE disease parameters, AID−/−μS−/− mice were statistically indistinguishable from WT mice in raw paralysis scores exhibited over time, day of disease onset, peak disease score, and cumulative disease index compared to their μMT counterparts (Fig. 4A, table). These data indicate that the absence of antibody production by B cells was insufficient to render mice resistant to EAE induction. To confirm immune responses in these mice, DTH was elicited by challenging the right ear pinnae with PLP_178–191_ and the left ear pinnae with PBS alone (similar to that described in Fig. 2 above) on day 23 post-immunization. As expected, given the clinical score data in Fig. 4A and DTH data in Supplementary Fig. 1, μMT mice exhibited an inferior DTH reaction compared to their B cell-sufficient counterparts, indicating that the lack of antibodies had little effect in driving inflammatory responses and EAE (Fig. 4B), and further supports that antigen-presenting cells other than B cells (eg. dendritic cells) are insufficient for optimal T cell priming in PLP_ECD_-immunized B6 mice.

## Conclusions

We present here a novel B cell-dependent, PLP-driven murine EAE model. These results indicate that PLP_ECD_ engages WT B6 B cells more efficiently than a short, albeit immunodominant peptide (PLP_178–191_), leading to a likely pathogenic antigen-presenting cell role. This may ultimately mimic what is occurring in human MS patients, and warrants further study into whether depletion of PLP_ECD_-driven B cells yields protection from demyelination. Any potential cellular and molecular immunological knowledge gained from this model may have increased translational implications given PLP_ECD_ utilizes sequences from the highly abundant (50% of total CNS myelin protein) and highly conserved (shares 100% amino acid sequence homology between mouse and human) myelin PLP. This work contributes an additional model to the field for investigating the pathogenesis and regulation of demyelinating disease, offering multiple ways for investigators to confirm immunological findings. PLP_ECD_ also provides an efficient way to investigate B cell-dependent demyelinating disease, as it obviates the need for various full-length protein expression systems in order to elude high titers of recombinant protein. It is also worth noting this study may represent a proof of principle that longer peptides (an 83-mer in this case) encompassing immunodominant sequences may be able to be designed for study of B cells’ role in other autoimmune diseases.

## Supplementary information

Supplementary Information.
