# Supplementary material for: Novel B cell-dependent multiple sclerosis model using extracellular domains of myelin proteolipid protein
Source: Sci Rep. 2020 Mar 19;10:5011. doi: 10.1038/s41598-020-61928-w (PMC7081236; doi:10.1038/s41598-020-61928-w)
Supplement: Supplementary file 1 — Supplementary Information. [file 41598_2020_61928_MOESM1_ESM.pdf]

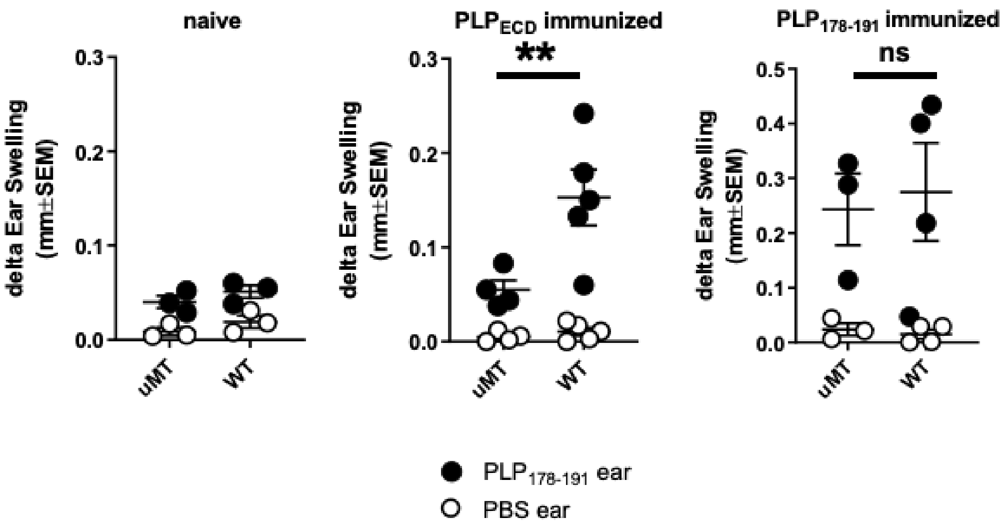

Supplementary Figure 1. PLP$_{178-191}$-driven DTH is impaired in PLP$_{ECD}$-immunized but not PLP$_{178-191}$-immunized μMT mice. Groups of WT and μMT mice were immunized s.c. with either 100μg of PLP$_{178-191}$/CFA or PLP$_{ECD}$/CFA on day 0. Twenty days later, naïve or immune mice were challenged with PLP$_{178-191}$ or PBS alone in right and left ear pinnae, respectively. Ears were measured at 48h. ns = not significant; ** $p < 0.01$.
